# Electrospun Nanofibers for Improved Angiogenesis: Promises for Tissue Engineering Applications

**DOI:** 10.3390/nano10081609

**Published:** 2020-08-17

**Authors:** Simin Nazarnezhad, Francesco Baino, Hae-Won Kim, Thomas J. Webster, Saeid Kargozar

**Affiliations:** 1 Tissue Engineering Research Group (TERG), Department of Anatomy and Cell Biology, School of Medicine, Mashhad University of Medical Sciences, Mashhad 917794-8564, Iran; smn.nazarnezhad@yahoo.com; 2 Institute of Materials Physics and Engineering, Applied Science and Technology Department, Politecnico di Torino, Corso Duca degli Abruzzi 24, 10129 Torino, Italy; 3 Department of Biomaterials Science, School of Dentistry, Dankook University, Cheonan 31116, Korea; kimhw@dku.edu; 4 Institute of Tissue Regeneration Engineering (ITREN), Dankook University, Cheonan 31116, Korea; 5 Department of Nanobiomedical Science & BK21 PLUS NBM Global Research Center for Regenerative Medicine Research Center, Dankook University, Cheonan 31116, Korea; 6 Department of Chemical Engineering, Northeastern University, 360 Huntington Avenue, Boston, MA 02115, USA; th.webster@neu.edu

**Keywords:** nanofibers, scaffolds, electrospinning, angiogenesis, tissue engineering, wound healing, nanotechnology

## Abstract

Angiogenesis (or the development of new blood vessels) is a key event in tissue engineering and regenerative medicine; thus, a number of biomaterials have been developed and combined with stem cells and/or bioactive molecules to produce three-dimensional (3D) pro-angiogenic constructs. Among the various biomaterials, electrospun nanofibrous scaffolds offer great opportunities for pro-angiogenic approaches in tissue repair and regeneration. Nanofibers made of natural and synthetic polymers are often used to incorporate bioactive components (e.g., bioactive glasses (BGs)) and load biomolecules (e.g., vascular endothelial growth factor (VEGF)) that exert pro-angiogenic activity. Furthermore, seeding of specific types of stem cells (e.g., endothelial progenitor cells) onto nanofibrous scaffolds is considered as a valuable alternative for inducing angiogenesis. The effectiveness of these strategies has been extensively examined both in vitro and in vivo and the outcomes have shown promise in the reconstruction of hard and soft tissues (mainly bone and skin, respectively). However, the translational of electrospun scaffolds with pro-angiogenic molecules or cells is only at its beginning, requiring more research to prove their usefulness in the repair and regeneration of other highly-vascularized vital tissues and organs. This review will cover the latest progress in designing and developing pro-angiogenic electrospun nanofibers and evaluate their usefulness in a tissue engineering and regenerative medicine setting.

## 1. Introduction

Therapeutic angiogenesis has been considered as a fundamental process for developing efficient tissue-engineered constructs, which facilitates the mass transfer of oxygen, nutrients, growth factors, signaling molecules, and metabolic waste from the extracellular milieu to cells, and vice versa [1,2]. As most cells in the body are found at a distance of 100–200 μm from the nearest capillary, oxygen, nutrients, and waste products reach them via the diffusion process. Therefore, successful tissue regeneration and replacement approaches require a highly vascularized network, reaching within 100–200 µm of cells to prevent an ischemic condition and tissue necrosis [3,4]. Therefore, tissue constructs should integrate a three dimensional (3D) interconnected capillary network to facilitate the repair and regeneration of human tissues (e.g., bone and skin). The initiation and ingrowth of a vascular network into transplanted tissues are followed by pro-angiogenic signaling pathways in which pro-angiogenic growth factors (mainly vascular endothelial growth factor (VEGF) and basic fibroblast growth factor (bFGF)) are major constituents. In particular, an ordered and sequential interaction happens between pro-angiogenic growth factors and extracellular matrix (ECM) formation, leading to a synergistic effect on both the adhesion and proliferation of endothelial cells [5,6,7,8,9]. In addition, the surrounding cells, including pericytes and smooth muscle cells (SMCs), in the angiogenic microenvironment also participate in the maturation and stabilization of newly formed vascular networks [10,11,12,13]. Table 1 summarizes some of the bioactive molecules (e.g., growth factors, cytokines, and so on) involved in the angiogenesis process and their functions in chronological order.

However, the use of transplanted materials carries a series of limitations (e.g., availability, risk of immune rejection [15,16]) and, therefore, attention has turned to formulate manmade implantable grafts [17]. Along with recent advancements in developing 3D scaffolds capable of promoting angiogenesis, electrospun nanofibers have gained much attention owing to their numerous advantages including its biomimetic in vivo ECM structure, high surface area to volume ratio, versatility in polymer selection (natural and synthetic types or their composites), easy process, and tunable integration with other scaffolds like hydrogels and 3D bio-printed constructs. Promoting angiogenesis by electrospun mats has been one of the most desirable therapeutic targets in which several researchers have globally tried to make angiogenic nanofibrous scaffolds and determine parameters that affect angiogenesis [18,19]. For example, it has been reported that electrospun scaffold architectures (pore size and fiber diameter) might modulate macrophage and mast cell responses, and thereby affect the secretion of VEGF [20,21]. Table 2 shows some parameters of fibrous scaffolds effective on the angiogenesis process.

It has been previously documented that nanofibrous scaffolds made of ECM-like filaments (e.g., collagen) could serve as suitable substrates for endothelial cell adhesion, proliferation, and migration, leading to the promotion of vascularization [30,31,32]. Furthermore, electrospun nanofibers have been widely used as suitable drug delivery vehicles for the sustained release of various angiogenesis-inducing growth factors, cytokines, and other bioactive molecules [33,34,35,36]. It should be noted that the co-delivery of angiogenic growth factors and other bioactive molecules by nanofibrous scaffolds is also suggested to improve tissue repair and regeneration [37].

In addition to growth factors and cytokines, electrospun nanofibers have been utilized to load and control the release of angiogenic small molecules (e.g., angiogenin) and phytochemicals (e.g., curcumin) in order to accelerate the healing process [38,39]. However, it should be highlighted that the angiogenic efficacy of such substances is strongly dose-dependent [40,41]. It is worth mentioning that surface functionalization of nanofibrous scaffolds by angiogenic bioactive molecules was also proven as a feasible approach for promoting tissue repair and regeneration [42,43]. Apart from these molecules, enhanced neovascularization could be achieved by adding inorganic elements (e.g., cerium and europium) to electrospun scaffolds [44,45,46]. Although there are several inorganic elements with the potential ability to induce angiogenesis, the risk of cytotoxicity and genotoxicity still limits their extensive use in tissue engineering applications [47]. In order to overcome this restriction, highly controlled doping of angiogenic elements to the structure of bioceramics (e.g., calcium phosphates and bioactive glasses) is suggested. In this sense, the incorporation of angiogenic metal-doped bioceramics into electrospun nanofibers sets up new possibilities for biomedical engineers to fabricate effective tissue substitutes [48,49].

From a biological point of view, different somatic and stem cells (e.g., endothelial cells (ECs) and mesenchymal stem cells (MSCs)) could be easily seeded onto nanofibrous scaffolds to promote angiogenesis, and subsequently accelerate the wound-healing process in injured sites [50]. This benefit originates from the inherent ability of cells to secret pro-angiogenic factors as well as to facilitate tubulogenesis (formation and expansion of the vascular lumen into newly formed branches) [51,52]. Moreover, the composition of electrospun mats may trigger and enhance the angiogenic activity of cells. For instance, nanofibrous scaffolds made of collagen-poly(ε-caprolactone) (PCL) and BG nanoparticles were used to efficiently deliver endothelial progenitor cells (EPCs) for enhancing wound healing. The incorporation of BG nanoparticles into the constructs may lead to accelerated wound healing as a result of enhanced cell viability and angiogenesis [53].

In the present review, we aim to introduce electrospun nanofibers as excellent 3D structures in angiogenesis-modulated tissue healing and highlight their potential as drug delivery systems. For this purpose, the key role of angiogenesis in tissue repair and regeneration is briefly presented, and then different scenarios applied to fabricate angiogenic electrospun nanofibrous scaffolds will be discussed. Finally, therapeutic applications of angiogenic nanofibers in both hard and soft tissues are introduced to determine their progress in tissue engineering and regenerative medicine. To the best of the authors’ knowledge, this review is the first of its kind dealing with the critical role of electrospun nanofibrous scaffolds in the regulation of angiogenesis and subsequent wound healing process critical for all tissue engineering applications.

## 2. Angiogenesis: A Critical Procedure in Tissue Engineering

Angiogenesis is defined as the process of sprouting new capillaries from pre-existing blood vessels, which occurs in sequential steps, including the following: (1) breakdown of the vascular basement membrane; (2) the expansion and alignment of ECs, which leads to lumen formation; (3) sprouting and migration via penetrating the ECM; (4) vessel maturation; and (5) stabilization [54,55,56]. After any injury to the body, the rupture of blood vessels appears as the most common event and their rebuilding is of great significance in the wound healing process. Therefore, biomedical engineers and biologists have focused on the development of functional tissue-engineered constructs to quickly re-establish blood flow to damaged tissues [57,58,59]. Indeed, blood supply provides oxygen, essential nutrients, signaling molecules, and growth factors to the injured sites, while removing waste by-products from the surrounding environment of cells. All of these events result in the improved viability of tissue-engineered constructs to prevent ischemic and necrotic conditions [60,61].

To date, numerous strategies have been exploited to promote neovascularization, including the fabrication of vascularized tissues [62], the incorporation of pro-angiogenic molecules into 3D constructs [63], and physicochemical treatment of scaffolds [64]. In this regard, the electrospinning of nanofibers has been proposed as an easy fabrication method to prepare angiogenic 3D constructs for accelerating the wound healing process.

## 3. Electrospun Nanofibers: A Brief Overview

In the past few years, impressive research has been directed towards electrospun nanofibers for numerous biomedical applications such as tissue repair and tissue regeneration as well as drug delivery [65]. Electrospinning provides the possibility of fabricating a wide variety of ECM-mimicking scaffolds with a high surface area and porosity by using both natural and synthetic polymers [66,67]. The basic components of an electrospinning system consist of (1) a high-voltage power supply, (2) a syringe with a metallic needle (to transfer the polymer solution), and (3) a metallic collector (to deposit nanofibers) [68,69,70]. In this process, a high voltage (in the range of 10–20 kV) is applied between the polymer solution in the syringe (as a positive electrode) and the collector plate (as a negative electrode), which leads to nanofiber formation by solvent evaporation from the jet [68,71,72,73,74].

It has been well understood that processing conditions applied by the electrospinning machine could influence the characteristics of the final produced electrospun fibers (mats) (Table 3). These situations could be summarized in specific groups including the electrospinning environment (e.g., humidity and temperature); electrospinning parameters (e.g., applied voltage (in the range of kV), needle tip to collector distance (cm), and the flow rate of the solution (µL/min)); and polymer solution properties like polymer molecular weight, solution viscosity, and solvent volatility [75,76,77,78,79]. For example, the fiber diameter increases not only owing to the enhanced concentration and higher molecular weight of the polymers used, but also by increasing the solution flow rate. However, the increase in other parameters for the polymers used such as electrical conductivity could lead to decreased fiber diameter [75].

According to the number and structure of the nozzles and polymers used, the electrospinning technique could be divided into two categories, including uniaxial and multiaxial electrospinning [89]. Uniaxial electrospinning is a simple and common method to fabricate a mixture of polymers and drugs, but it lacks the potential of achieving a finely-controlled drug release rate [90]. On the contrary, it is possible to produce core-shell nanofibers with enhanced drug release efficacy (the drug is loaded into the core) using coaxial electrospinning [91,92]. In the last decade, triaxial electrospinning has motivated researchers to fabricate triaxial nanofibers with a highly controlled release profile. In this context, a three-layered electrospun mat could be constructed with the same polymeric composition, but different drug content, which generates a gradient distribution of active ingredients [93]. In addition, multiaxial electrospinning allows for delivering multiple therapeutic agents with different release time profiles [94]. The structure and shapes of the produced mats could be divided into multilayer [95], hollow [96,97], side-by-side [98], twisted [99,100], and porous surface nanofibers [101,102]. It is worth mentioning that different shapes may affect the physicochemical and functional properties of final product. Moreover, surface modification of the nanofiber mats (e.g., covalent and non-covalent immobilization and plasma treatment) is also considered as a critical parameter in determining the physico-chemical and biological properties like drug delivery efficiency [68,103,104]. On the basis of the desired applications, a broad range of raw materials could be used to fabricate electrospun mats, including natural polymers (e.g., collagen, chitosan, alginate, and hyaluronic acid), synthetic polymers (e.g., PCL, poly(lactic acid) (PLA) and poly(lactic-co-glycolic acid) (PLGA)), and their combinations [75]. It is worth noting that the development of electrospun nanofiber-reinforced composites is being currently studied to further improve the physico-chemical and biological properties of the final construct [105].

## 4. Electrospun Nanofibers Meet Angiogenesis

**Figure 1:**
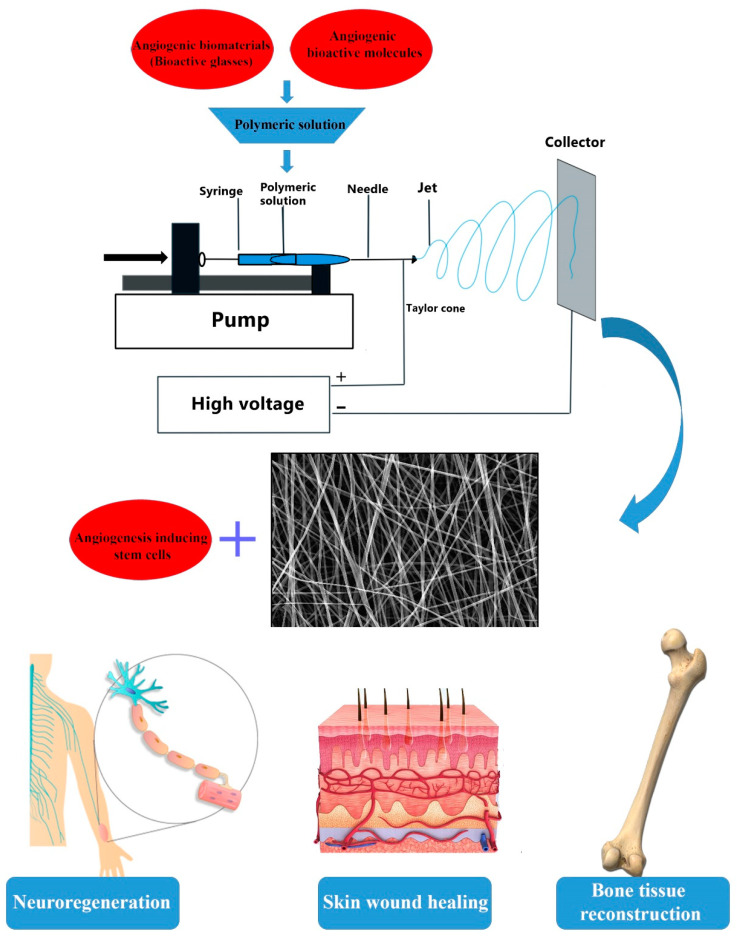
Neo-vessel formation can be induced by applying electrospun nanofibers containing pro-aniogenic bioactive molecules (e.g., vascular endothelial growth factor (VEGF)), angiogenesis inducing progenitor/stem cells (endothelial progenitor cells, EPCs), and angiogenic biomaterials (e.g., bioactive glasses). The fabricated angiogenic nanofibers could be applied for accelerating wound healing process for both hard (e.g., the bone) and soft tissues (e.g., the skin and the peripheral nerves).

Recent evidence has revealed that enhanced angiogenesis can be achieved by applying nanofibrous mats. As depicted in Figure 1, angiogenic inducing electrospun mats could be easily fabricated by adding specific materials (e.g., bioactive glasses (BGs)) and bioactive molecules (e.g., VEGF) to polymeric substrates. In addition, seeding progenitor/stem cells (e.g., EPCs) onto fibrous scaffolds has been proposed as another reasonable approach regarding enhanced neo-vessel formation. It has been identified that inherent characteristics of fibers including pore size, porosity, and alignment are key determinants for the outcome [106]. For example, small pore size is a major obstacle for cell infiltration and vascularization. The pore size of electrospun nanofibers can be modulated by properly acting on electrospinning parameters and/or by combining electrospinning with gas foaming, salt leaching, and electrospraying [107]. According to the literature, a pore size of 300 µm in the nanofibrous scaffolds has been identified as an optimal dimension to induce new vessel formation [108].

Prior experimental studies have confirmed the intrinsic angiogenic properties of some natural and synthetic polymers (e.g., collagen [109], elastin [110], and PLA [111]) and their composites (Table 4). As an illustration, hyaluronic acid oligosaccharide-modified collagen nanofibers have been proven to promote the proliferation and endothelialization by ECs [112]. However, scientific evidence is limited in the case of angiogenic natural polymers, and experimental studies on synthetic polymers are fairly more abundant than those with naturally-derived polymers. PCL-based electrospun nanofibers are among the most used nanofibrous mats as angiogenic biomaterials in different regenerative models including choroidal neovascularization [113], artery substitute for long-term implantation [114], and smooth muscle tissue [115]. Moreover, PCL copolymers such as poly(lactide-co-ε-caprolactone) (PLCL) have been shown to induce angiogenesis after implantation in a rat model, in which more and larger vessels were formed and penetrated the construct after intraperitoneal implantation compared with subcutaneous implantation [116]. It should be pointed out that other PCL-based constructs (e.g., nanofiber-hydrogel composites) have also shown the ability to induce angiogenesis. For instance, Li et al. produced nanofibrous PCL-hyaluronic acid hydrogel composites that mimicked the structure and mechanical properties of the soft tissue ECM. This network allowed for the infiltration of macrophages and guided them to the regenerative phenotype, which ultimately led to the promoted expression of pro-angiogenic growth factors and cytokines including VEGF-D, matrix metalloproteinase-2 (MMP2), matrix metalloproteinase-3 (MMP3), and matrix metalloproteinase-19 (MMP19) after subcutaneous injection in rat and rabbit models [117]. Another synthetic polymer that was shown to have pro-angiogenic properties is poly(L-lactic acid) (PLLA) [118]. PLLA could stimulate the migration of vascular smooth muscle cells (VSMCs) via an NF-κB-dependent pathway, which in turn could promote angiogenesis [119].

The use of electrospun nanofibrous composites based on a combination of both natural and synthetic polymers has also been examined to promote angiogenesis. As an illustration, Gugutkov et al. fabricated composite scaffolds of electrospun fibrinogen (FBG)-PLA to take advantage of the excellent cell recognition properties of native FBG and biomechanical properties of PLA [26]. The obtained data revealed that the aligned nanofibers promoted an elongated EC shape and enhanced cell mobility, leading to faster wound coverage, while the stellate-like morphology of the ECs was observed on the random nanofibers. In addition, the results of a nitric oxide (NO) release assay showed that human umbilical vein endothelial cells (HUVECs) possessed increased functionality on random nanofibers as compared with the aligned counterparts. The authors concluded that random nanofibers might support endothelialization, whereas aligned ones could guide neovascularization of implants.

### 4.1. Nanofibers as Delivery Systems of Angiogenic Substances

Electrospun nanofibers benefit from a high loading efficiency of therapeutic agents owing to a large surface area to volume ratio compared with other conventional nanoscale delivery vehicles like liposomes, polymeric micelles, and complexes. Furthermore, the tunable and tailored properties of the polymer matrix (e.g., porosity, diameter, and morphology) provide the possibility of incorporating various drugs into electrospun scaffolds, thus leading to effective localized delivery of drugs to the targeted tissue [125]. Several methods have been developed to enhance the encapsulation efficiency of therapeutic agents into the nanofibrous mats, hindering their burst release, including pre-electrospinning (blending, emulsion, and coaxial electrospinning/electrospraying) as well as post-electrospinning methods (chemical immobilization, physical adsorption, and layer-by-layer assembly) [126,127]. To date, a huge number of attempts have been made to create pro- and anti-angiogenic nanofibrous mats for potential use in a wide range of biomedical applications, from cancer therapy to tissue engineering and regenerative medicine. However, the main concern of the following section is to present and discuss the most important experimental studies concerning angiogenic nanofibrous mats in either pristine or modified forms (different types of chemicals, bioactive molecules, cells, as well as biomaterials), which can facilitate the generation of vascularized tissue-engineered constructs.

#### 4.1.1. Pro-Angiogenic Growth Factor/Hormone-Loaded Nanofibrous Scaffolds

Encapsulation of pro-angiogenic growth factors into electrospun nanofibers is an effective and direct strategy to promote angiogenesis in different physiological and pathological conditions. To date, a large number of experimental studies have successfully encapsulated pro-angiogenic growth factors (mainly VEGF) into nanofibrous mats by means of different techniques, such as coaxial electrospinning, to achieve sustained release profiles [128,129,130,131]. For example, in order to accelerate endothelialization along the lumen of graft, composite grafts were fabricated by co-electrospinning of chitosan hydrogel/polyethylene glycol (PEG)-b-poly(L-lactide-co-ε-caprolactone) (PLCL) loaded with VEGF as the inner layer and platelet-derived growth factor (PDGF)-loaded emulsion/PLCL nanofibers as the outer layer. Four weeks after construct and engraftment into the rabbit carotid artery, vascular endothelial cells (VECs) and vascular smooth muscle cells (VSMCs) were observed on the lumen and exterior of the vascular grafts, respectively [132]. In another study, genipin-crosslinked electrospun gelatin mats and were immersed in a human VEGF-containing solution (50 ng/mg dry mat, 5 mL/mg of dry mats) to stimulate early angiogenesis in vitro and in vivo [34]. The results proved the bioactivity and pro-angiogenic capacity of VEGF, which was retained for up to 14 days.

On the basis of the literature, it can be noticed that the simultaneous incorporation of multiple pro-angiogenic growth factors into electrospun nanomats may accelerate the angiogenesis process [132,133]. The pro-angiogenic factors VEGF [134,135], bFGF [136,137,138], and PDGF-BB [139] proved to recapitulate the in vivo physiological conditions, leading to functional and mature blood vessel formation. For instance, VEGF triggers the growth and proliferation of endothelial cells [140]; modulates the vascular permeability [141]; and, in combination with bFGF, induces the recruitment of endothelial cells, while PDGF is responsible for new vessel stabilization [142]. For instance, Lai et al. fabricated an electrospun construct consisting of collagen/hyaluronic acid nanofibers with a programmable release (up to one month) of VEGF, PDGF (with final concentration of 0.2 µg/µL, which was adsorbed in 10 mg gelatin nanoparticles for both VEGF and PDGF), bFGF, and EGF either embedded in the nanofibers or encapsulated in the gelatin nanoparticles to accelerate the wound closure rate [133].

It should be highlighted that innovative techniques, such as the combination of electrospinning with electrospraying, have also been utilized to deliver growth factors [126]. In this context, DeVolder et al. developed VEGF-encapsulated PLGA microparticles (10 µg/mL of VEGF), which were electrosprayed simultaneously with electrospun PLA fibers. This approach resulted in a sustained release of VEGF from PLGA microparticles, leading to a larger number and size of mature blood vessels (according to the CAM assay after 1 week), while the aligned PLA fibers without VEGF-encapsulated PLGA microparticles guided the orientation of new blood vessel formation [143].

It has been well demonstrated that hormones can mediate angiogenesis either by action on endothelial cells or through regulating pro-angiogenic factors like VEGF [144]. In this sense, PCL nanofibrous mats containing the triiodothyronine (T3) hormone were fabricated and showed an increased rate of endothelial cell proliferation, migration, and angiogenesis [145]. Heparin is an anticoagulation hormone that triggers the proliferation of HUVECs [146]. In order to promote the blood compatibility of nanofibers, coaxial electrospun scaffolds were constructed in which the inner layer was comprised of PLGA/collagen nanofibers modified by mesoporous silica nanoparticles-grafted with PEG and heparin and the outer layer was composed of polyurethane nanofibers to improve mechanical properties. This composite enhanced the proliferation of both endothelial cells and smooth muscle cells by immunostaining of CD31 and alpha-smooth muscle actin (α-SMA) markers after a rabbit carotid artery implantation, respectively [147]. In addition, heparinized nanofibrous scaffolds also showed the ability to promote the proliferation of ECs, and were thus proposed for the production of bioengineered blood vessels [148].

#### 4.1.2. Nanofibers Incorporating Phytochemicals and Other Bioactive Molecules

Phytochemicals form a major part of organic materials applied in tissue engineering and regenerative medicine [40,149,150,151,152]. Experimental data showed the pro-angiogenic capacity of some specific types of phytochemicals; for example, icariin is able to induce angiogenesis through activating the MEK/ERK- and PI3K/Akt/eNOS-dependent signal pathways in human endothelial cells [153]. However, it should be highlighted that some of the phytochemicals (e.g., curcumin) showed pro- or anti-angiogenic activities in a dose-dependent manner [154,155,156]. To date, a series of studies have shown the usability of phytochemical-incorporated electrospun nanofibers regarding pro-angiogenic strategies [43,157]. On the basis of the literature, there is still a scientific gap in this area and researchers are suggested to pay more focus to phytochemical-incorporated electrospun nanofibers for inducing angiogenesis.

Other bioactive molecules can also trigger angiogenesis including metallic ions, platelet-rich plasma (PRP) [158,159], ECM derivatives, recombinant proteins [160,161,162,163], and decellularized matrices [164]. Plenty of experimental studies have been conducted to survey the feasibility of such approaches to promote angiogenesis. For example, the effectiveness of copper impregnation into polymeric matrices (9 wt% and 4 wt% of CuSO_4_ in dimethylformamide with a polyurethane/copper volume ratio of 8:1 and 8:0.5 (*v*/*v*%) in two separate studies, respectively) when making the composite electrospun scaffolds clearly proved pro-angiogenic strategies [165,166]. In the case of natural components, increased secretion of VEGF and bFGF proteins was observed by applying a 3D bi-layer scaffold of a decellularized human amniotic membrane and electrospun nanofibrous silk fibroin seeded with adipose tissue-derived MSCs [167].

### 4.2. Bioactive Glass- and Bioceramic-Incorporated Electrospun Scaffolds

There is sufficient scientific evidence on the pro-angiogenic capacity of glasses, glass-ceramics, and calcium phosphates (CaPs) [168,169,170,171,172,173]. The main reason is attributed to the release of pro-angiogenic ions (e.g., Si^4+^) from BGs and glass-ceramics to the surrounding environment [174]. It should be emphasized that the incorporation of metallic elements (e.g., Cu^2+^ and Co^2+^) into BGs and glass-ceramics is a common approach to enhance their angiogenic potential [175,176]. Previously, the fabrication of electrospun nanofibers from strontium- and copper-doped BGs has been reported as a promising approach in regards to improving neovascularization [177]. In addition, the usability of 3D composite nanofibrous scaffolds made of glasses and polymeric matrices has been examined for pro-angiogenic strategies [178,179,180,181]. Differentiation of human endometrial stem cells (EnSCs) into endothelial-like cells was previously reported using gelatin/chitosan/bioactive glass (GEL/CS/BG) nanofibrous scaffolds [182]. In the presence of FGF-2 and VEGF, EnSCs differentiated into ECs and then were cultured onto the glass-containing scaffolds. The nanofibrous scaffolds were prepared by adding 0.5, 1.5, and 3% wt of BG nanoparticles (BGNPs) to a GEL/CS solution; the diameter of the obtained nanofibers increased along with reducing the BG content in the GEL/CS scaffolds. The cellular experiments showed that the nanofibrous scaffolds with 1.5% BGNPs were more suitable substrates for EC differentiation and possible use for blood vessel regeneration applications. In another study, PLA nanofibers containing calcium phosphate ormoglass particles showed the ability to promote angiogenesis via up-regulation of pro-angiogenic factors VEGF, insulin-like growth factor-2 (IGF-2), Fas ligand, granulocyte-macrophage colony-stimulating factor (GM-CSF), interleukin-1β (IL-1β), IL-6, and IL-12p70 in mammalian cells [183]. Apart from the mentioned studies, the experimental research on BG-incorporated electrospun nanofibrous scaffolds is indeed limited, and more investigations should be performed to reveal the pros and cons of BG-containing polymeric nanofibers for angiogenic promoting strategies.

CaPs were previously reviewed and introduced as materials having the potential to influence angiogenesis [169]. Therefore, the incorporation of CaPs into polymeric electrospun nanofibers is currently under investigation in the context of angiogenesis. As the biological behaviors of CaPs could be easily modified by adding trace metal ions to their structure, several studies have tried to enhance the angiogenic potential of CaPs by replacing therapeutic pro-angiogenic ions [184,185,186,187]. Ye et al. could enhance osteogenesis and angiogenesis by the incorporation of 20 wt% bioactive strontium-doped CaP nanoparticles into PCL/chitosan electrospun nanocomposite membranes via a one-step electrospinning method [188]. They reported that the nanocomposite membranes possessed similar structural properties of the native ECM and were able to support the adhesion, proliferation, and angiogenic differentiation of rat bone marrow mesenchymal stem cells (BM-MSCs) via enhancing VEGF secretion levels. Similar to the BG-incorporated electrospun nanofibrous scaffolds, more research is needed to determine which formulations may result in the best output and how much CaPs should be added to the nanofibrous scaffolds.

### 4.3. Cell-Laden Nanofibers for Pro-Angiogenesis Strategies

**Figure 2:**
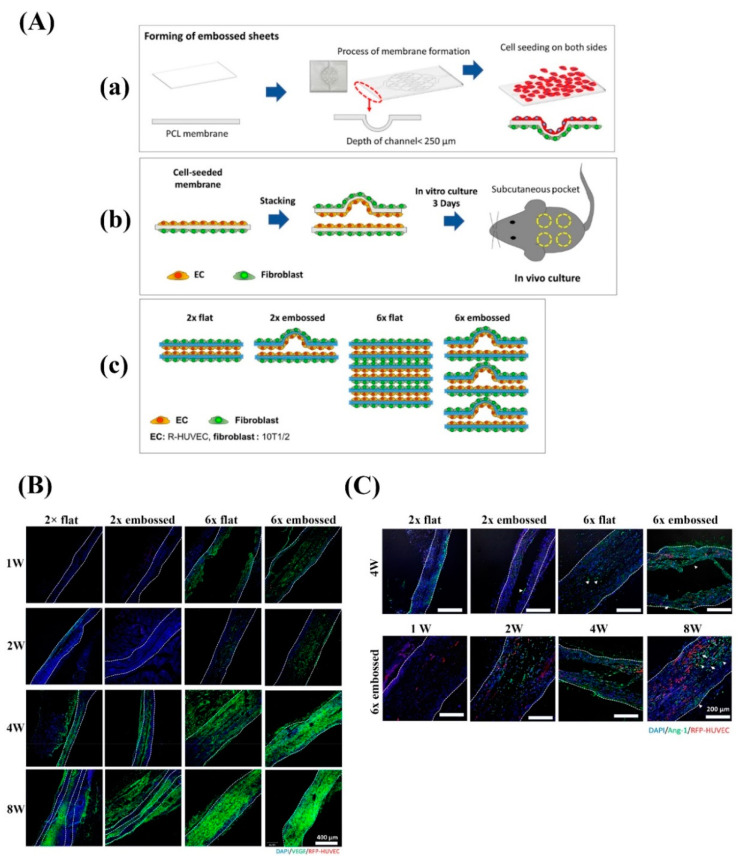
(**A**) A schematic representation of the preparation of electrospun nanofibrous membranes with embossed patterns for guided vascularization: (**a**) the formation of embossed sheet and plating of human ECs and mouse fibroblast cells, (**b**) stacking and incubating of the cell-laden sheets for 3 days before the implantation into subcutaneous pockets in mice, and (**c**) four different experimental groups evaluated in vivo. (**B**) Immunofluorescence images exhibiting differences in VEGF expression in different groups after 1, 2, 4, and 8 weeks of surgery; dashed lines show the edge of transplanted embossed sheets. Green: VEGF, red: human umbilical vein endothelial cell (HUVEC), blue: DAPI. (**C**) Immunofluorescence images exhibiting differences in angiopoietin 1 (Ang-1) expression in cross-sections of different groups after 1, 2, 4, and 8 weeks of surgery; dashed lines show the edge of transplanted embossed sheets. Green: Ang-1, red: HUVEC, blue: DAPI (adapted from Hong et al. [204]). PCL, poly(ε-caprolactone); EC, endothelial cell.

Culturing of stem cells onto nanofibrous scaffolds might result in enhanced angiogenesis; in general, various types of stem cells have been used to promote the angiogenic capacity of nanofibrous electrospun mats [189,190,191]. Endothelial progenitor cells, endothelial cells, mural cells like pericytes and smooth muscle cells, mesenchymal stem cells, and hematopoietic stem cells are among the most promising candidates for promoting angiogenesis as they are able to secrete pro-angiogenic growth factors and cytokines [192,193,194,195]. Regarding the outcome of numerous experimental studies, it can be stated that extracellular vesicles and exosomes containing pro-angiogenic growth factors are responsible elements for improving neovascularization [196,197,198,199]. For instance, extracellular vesicles released from human adipose-derived stem cells have been shown to promote angiogenesis through the let-7/AGO1/VEGF signaling pathway in an ischemic model of mice [198]. Despite this potent pro-angiogenic capacity, there is limited evidence of experiments in which pro-angiogenic exosomes and vesicles incorporated into the nanofibrous mats were shown to induce neovascularization. However, the encapsulation of various stem cells and EPCs secreting pro-angiogenic vesicles in electrospun nanofibers has been regarded as an efficient strategy to accelerate neovascularization in various tissues [200,201,202]. In this sense, EPCs were able to induce tubular structure formation into a highly porous 3D electrospun scaffold composed of PCL. The large pore size (pore size > 400 µm) of this scaffold regulated EPC behavior, that is, cell infiltration, proliferation, colonization, and new blood vessel formation [106]. Previously, the co-culturing of EPCs with perivascular cells like MSCs or fibroblasts has been proposed as a good strategy to make long-lasting and functional vascular networks [203]. In this sense, Hong et al. fabricated six-layered PCL nanofibrous constructs (12.5% w/v) and seeded both sides of each layer with endothelial cells and pericyte cells (Figure 2). The results obtained from confocal microscopy showed an embossed vascular pattern (208.8 ± 44.5 µm in height) up to 3 days post-cell culture. New blood vessel formation and maturation were also confirmed by the upregulation of VEGF and Ang-1 genes in the 6x embossed group [204]. Furthermore, co-culturing of ECs with bone and adipose tissue derived mesenchymal stem cells (BM-MSCs and AD-MSCs) led to promoted endothelial tubulogenesis [205].

Some studies also revealed the applicability of BM-MSCs- and AD-MSCs-loaded nanofibrous scaffolds in promoting angiogenesis [206,207,208,209]. With the rise of novel cell technologies, such as the methods to manipulate induced-pluripotent stem cells (iPSCs), researchers are currently able to generate iPSC-derived endothelial cells (iPSC-ECs) as a promising source of ECs for therapeutic angiogenesis [210,211,212]. However, there are a few reports in the literature concerning the in vivo engraftment of iPSC-ECs for boosting neovascularization. Tan et al. showed that the incorporation of iPSC-ECs into electrospun PCL/gelatin scaffolds could increase the pro-angiogenic function via enhanced expression of VEGF and placental growth factor (PLGF) [213]. Despite recent advancements of iPSCs in the field of regenerative medicine, a lack of investigation still remains a pressing issue for the use of this cell type in angiogenesis-stimulant nanofibrous mats.

Table 5 summarizes a list of experiments in which various cells/nanofibrous scaffolds were applied to improve angiogenesis. As electrical stimulation can promote the angiogenesis in different tissues and cell types (including the artery [214], skeletal muscle [215], peripheral nerve [216], wound healing [217,218], endothelial cells [219,220], human mesenchymal stromal cells [221], adipose tissue-derived stem cells [222], cardiomyocytes [223], and osteoblasts [224]), it may be useful to use electrospun conducting polymer nanofibers to obtain better in vitro and in vivo results in future investigations. However, there is some evidence of using conductive polymers in hydrogel constructs with pro-angiogenic potential (such as N-carboxyethyl chitosan and oxidized hyaluronic acid-graft-aniline tetramer [225], gelatin-grafted-dopamine and polydopamine-coated carbon nanotubes [226], and polypyrrole [227]), but there is a lack of research on such polymers incorporated in electrospun nanofibers with a pro-angiogenic approach.

## 5. Angiogenic Nanofibrous Scaffolds in Tissue Engineering

The fabrication and use of pro-angiogenic electrospun mats are of great interest in tissue engineering approaches regarding their ability to accelerate the wound healing process. Therefore, several studies have tried to implement angiogenic nanofiber mats for the reconstruction of both hard and soft tissues. In the following sections, we summarize and discuss the angiogenic electrospun nanofibrous scaffolds in the frame of tissue engineering and regenerative medicine. The reviewed studies are also collected in Table 6.

### 5.1. Angiogenic Nanofibers for Hard Tissue Engineering

**Figure 3:**
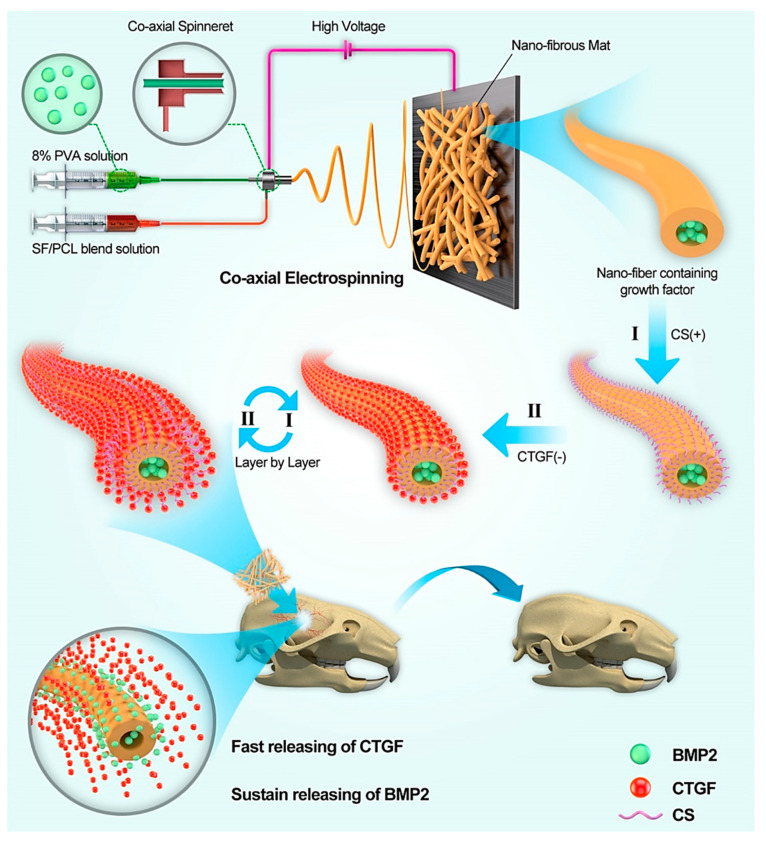
Schematic illustration of the bone healing process via controlled co-delivery of connective tissue growth factor (CTGF) and bone morphogenic protein-2 (BMP2) by combining the technique of coaxial electrospinning and layer-by-layer (LBL) process. BMP2 and CTGF were incorporated and attached to the core and the surface of the nanofibers, respectively (adapted from Cheng et al. [246]). SF: silk fibroin and CS: chitosan.

The fundamental role of therapeutic angiogenesis has been well established during the repair and regeneration of hard tissues like bones. Local delivery of osteogenic and angiogenic growth factors (mainly bone morphogenic protein-2 (BMP2), FGF, and VEGF) via electrospun nanofibrous membranes were considered as a promising strategy for overall enhanced osteogenesis [242], in which, specifically, BMP2 enhances osteogenic differentiation of MSCs [243], VEGF promotes angiogenesis of MSCs [244], and bFGF promotes cell proliferation and migration as well as tube formation of HUVECs [245]. It is worth noting that the sequential release of various growth factors from the electrospun nanofibers could accelerate vascularized bone formation. Accordingly, Cheng et al. fabricated multilayer core-shell silk fibroin (SF)/PCL/PVA nanofibrous scaffolds containing BMP2 (10 µg/mL) and connective tissue growth factor (CTGF) (10 µg/mL) [246]. For this aim, they incorporated BMP2 into the core of the mats by applying coaxial electrospinning and then immobilized CTGF onto their surface via a layer-by-layer (LBL) self-assembly technique (Figure 3). The efficacy of the prepared scaffolds was examined in vitro and in vivo to reveal their potential in bone tissue engineering applications, with a special focus on the promotion of osteogenesis and angiogenesis. The in vitro results revealed a sustained and linear release profile for BMP2 (during 30 days) and a burst release for CTGF (during 40 days). The results of the implantation of the nanofibrous mats into a critical-size cranial defect of mice models revealed a significantly higher newly-formed bone percentage (43%) in the animals treated with the BMP2/CTGF-loaded (SF/PCL) 1:5/PVA-LBL_20_ scaffolds in comparison with the (SF/PCL) 1:5/PVA nanofibrous mats loaded with BMP2 alone after 4 weeks. The same trend was observed in the samples harvested at 8 weeks post-implantation in mice as the largest bone area and bone volume belonged to the BMP2/CTGF-loaded (SF/PCL) 1:5/PVA-LBL_20_ nanofibrous mats. Furthermore, angiogenic markers of VEGF and CD-31 were over-expressed in the animals treated with the BMP2/CTGF-loaded (SF/PCL) 1:5/PVA-LBL_20_ group. The authors concluded that sequential and dual-delivery of BMP2 and CTGF might be useful in inducing osteogenesis and angiogenesis, ultimately yielding accelerated bone formation with new blood vessels.

There are limited reports in the literature dealing with the application of bioceramic-containing polymeric nanofibrous mats for promoting angiogenesis and subsequently accelerating bone tissue healing. In this content, Oliveira et al. developed injectable composite scaffolds made of Ca-/P rich ormoglass particles prepared by a partial hydrolyzed alkoxides sol-gel method with a composition of 44.5:44.5:6:5 of CaO:P_2_O_5_:Na_2_O:TiO_2_ molar ratios dispersed in (hydroxypropyl) methyl cellulose (HPMC) gels (40% *w*/*v*) coated with PLA fibers [247]. They evaluated the osteogenic and angiogenic capacities of the constructs in femoral condyles of Wistar rats. The release of Ca^2+^ ions from the scaffolds had a positive impact on bone formation and angiogenesis after 3 and 6 weeks of implantation.

**Figure 4:**
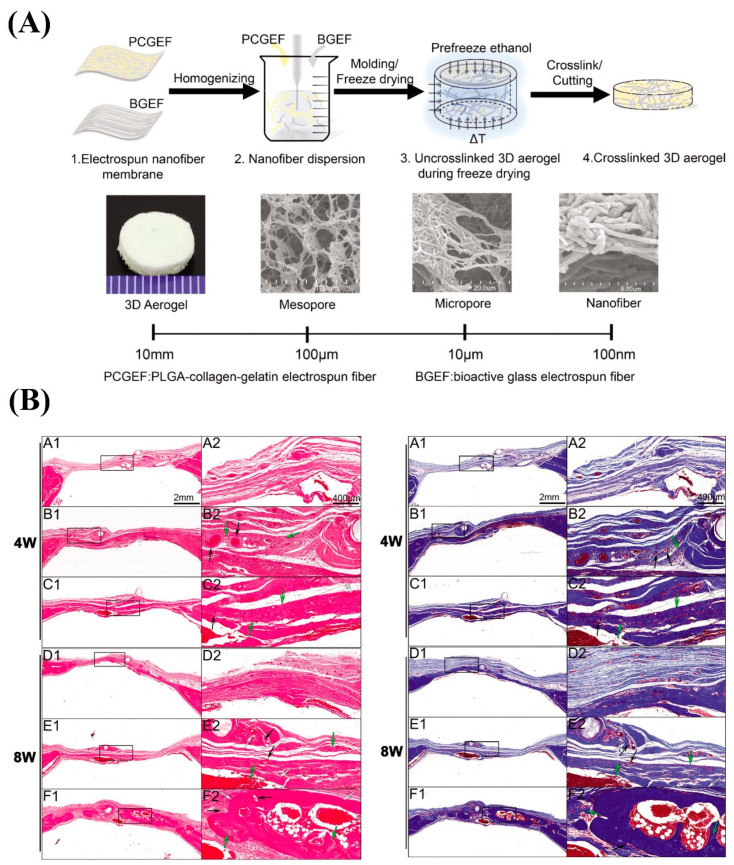
(**A**) Schematic representation of different steps of the preparation of the 3D hybrid nanofiber aerogels and its structure. (**B**) Images of hematoxylin and eosin-stained (left) and Masson’s trichrome-stained (right) tissue slides at 4 and 8 weeks post-implantation into critical-sized calvarial defects in rats. (A1/A2, D1/D2) Unfiled defects; (B1/B2, E1/E2) the defects implanted by 3D hybrid nanofiber aerogels (PCG/BG = 60:40); and (C1/C2, F1/F2) the defects implanted by E7-BMP-2 peptide-loaded 3D hybrid nanofiber aerogel (PCG/BG = 60:40) (black arrow: blood vessel in aerogel; white arrow: aerogel residue; green arrow: new bone) (adapted from Weng et al. [249], with permission from John Wiley and Sons).

As the incorporation of cytokines and bioactive molecules (e.g., BMP2) into electrospun mats may have adverse effects on their bioactivity, researchers have proposed the physical adsorption method as a gold standard to retain bioactivity [248]. In this regard, 3D hybrid nanofiber aerogels were fabricated with a formulation of PLGA-collagen-gelatin (PCG) and Sr/Cu co-doped BGs fibers incorporated with E7 domain-specific BMP-2 peptides [249]. Sr^2+^ and Cu^2+^ ions were added to the glass to promote neo-bone formation and vascularization, respectively. The degradable hybrid aerogels were implanted into critical-sized defects (8 mm in diameter) created in rat calvaria to determine its efficacy in the bone healing process. Data obtained from radiography and histopathological assessments revealed a 60–70% closure of critical-sized defects in the injured sites (Figure 4).

A critical issue in bone tissue engineering is osteoclast-mediated bone resorption and osteoblast-mediated bone formation in the process of bone remodeling [250]. In 2019, Wang et al. developed mesoporous silicate nanoparticle (MSN)-based electrospun PCL/gelatin nanofibers for the dual delivery of alendronate (ALN) and silicate ions [251]. The concept behind this study was to achieve a synergetic effect in modulating bone remodeling because ALN could inhibit the bone-resorbing process through inhibiting of guanosine triphosphate-related protein expression, while silicate ions promote the bone-forming process by promoting angiogenesis and bone calcification. The release of both ALN and silicon (in the form silicate ions) from the scaffolds was observed, indicating the success of dual drug delivery using the scaffolds. The results of in vivo implantation of the ALN@MSN-loaded nanofibers in a rat critical-sized cranial defect model revealed an accelerated bone healing time (from 4 weeks to 12 weeks post-implantation), which was three times faster in comparison with the bare scaffolds.

### 5.2. Angiogenic Nanofibers for Soft Tissue Regeneration

#### 5.2.1. Angiogenic Nanofibrous Mats for Skin Regeneration

Angiogenesis plays a pivotal role in soft tissue wound healing, especially in the case of chronic wounds [252,253]. Several electrospun membranes have shown great abilities to improve the healing process of epidermal and dermal layers [163,254]. By highlighting the role of angiogenesis in the skin regeneration process, a multifunctional and biomimetic nanofibrous membrane was composed of fish collagen type I (COL) and bioactive glasses (BGs) using the electrospinning method [255]. The prepared nanofibers (ratio of COL/BG was 10:1) had a fiber diameter of 494 ± 193 nm and could not only promote the adhesion and proliferation of HaCaT cells, but also upregulate the expression of TGF-β and MMP-9 genes. In addition, dermal regeneration and angiogenesis were also promoted by COL/BG nanofibrous mat regarding the increased adhesion and proliferation of human dermal fibroblasts (HDFs) (*p* < 0.05) as well as secretion of collagen type I protein and VEGF (*p* < 0.05). The histological results obtained from the cutaneous implantation of COL/BG nanofibers into rats revealed a higher formation of collagen fibers and rapid re-epithelialization and angiogenesis in the injured sites.

Diabetic wounds usually suffer from an impaired wound healing process owing to the vascular impairment resulting from the delayed secretion of pro-angiogenic factors as well as reduced proliferation and migration of ECs and tissue re-epithelialization [255,256,257,258]. In this regard, many researchers have made significant attempts to develop an efficient pro-angiogenic wound dressing for accelerating diabetic wound healing and tackling this important societal and clinical challenge [133,259]. In 2018, Ren et al. reported the successful fabrication of electrospun fibrous membranes made of poly(L-lactic acid) (PLLA) containing dimethyloxalylglycine (DMOG)-loaded mesoporous silica nanoparticles (DS) for potential usage in diabetic wound healing [260]. This system could act as a sustained release vehicle for DMOG and silicon ions, leading to stimulating the proliferation, migration, and angiogenesis-related gene expression of HUVECs in comparison with the PLLA membranes. The in vivo implantation of the constructs in the dorsal skin of diabetic mice showed an improvement in neo-vascularization, re-epithelialization, and collagen formation 15 days post-surgery.

Electrospun nanofibers have been previously used for the encapsulation and sustained release of pro-angiogenic growth factors to accelerate skin wound repair [261]. Conventional efforts have relied on the LBL assembly and coaxial electrospinning to achieve the controlled release of multiple growth factors in which the diffusion between layers is a major hindrance [262,263,264]. In this regard, the use of nanoparticles is suggested to obtain a more effective sustained-release [261]. It has been previously reported that neovascularization within the wound site might be improved by applying a nanofibrous scaffold composed of collagen, PCL, and bioactive glass nanoparticles (CPB) seeded with EPCs [53]. The mechanism proposed for this improvement was attributed to the activation of the HIF-1α/VEGF/SDF-1α signaling pathway in the injured site, which can result in promoted cell proliferation, granulation tissue formation, and collagen synthesis and deposition.

As an outstanding study, a research team under the supervision of Prof. Wu reported simultaneous tumor therapy and skin wound regeneration by applying electrospun micro-patterned nanocomposites incorporated with Cu_2_S nanoflowers [265]. Indeed, this strategy seems to be really suitable for effectively killing of the remaining tumor cells after surgical excision of skin tumors. The authors took benefits from the photothermal capacity of Cu_2_S nanoflowers and the angiogenic potential of Cu^2+^ ions in a substrate of poly(D, L-lactic acid)/PCL. The composite membranes (Cu_2_S incorporated PDLLA/PCL (CS-PLA/PCL) membranes) were produced using the patterning-co-electrospinning method, which could improve the adhesion, proliferation, and angiogenesis of ECs in vitro as well as accelerate in vivo wound healing. In addition, the hyperthermia induced by laser-irradiated 30CS-PCL membranes allowed for the ablation of skin tumor cells (B16F10 cells and A375 cells) in vitro and inhibited B16F10 tumor growth in vivo.

#### 5.2.2. Angiogenic Fibers for Neuroregeneration

**Figure 5:**
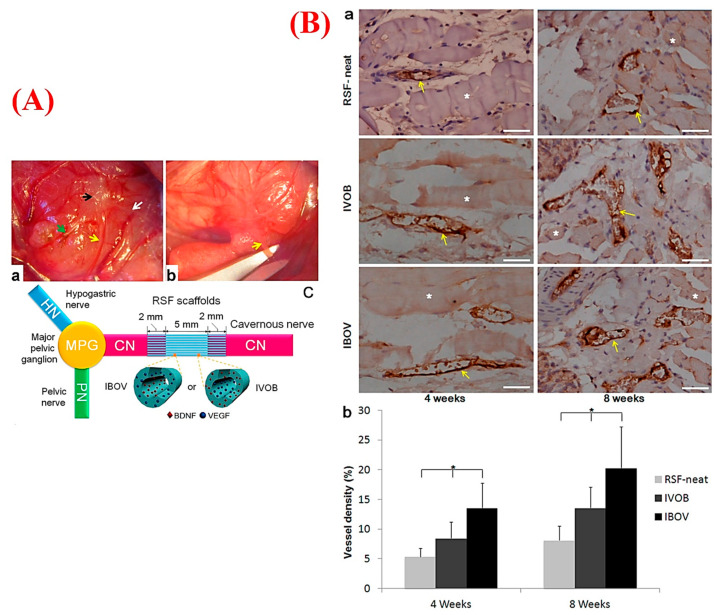
(**A**) Macroscopic views representing the implantation of regenerated silk fibroin (RSF) scaffolds into a rat animal model: (a) exposed major pelvic ganglion (MPG), pelvic nerve (PN), hypogastric nerve (HN), and cavernous nerve (CN) are marked as black, green, white, and yellow arrows, respectively; (b) creating the CN gap (arrow) (length of 5 mm) by scissors; and (c) schematic illustration of suturing process of RSF scaffolds and two CN ends. (**B**) Immunohistochemistry (IHC) staining of the harvested samples for evaluating new vessels in the retrieved scaffolds of RSF-neat, inner-VEGF/outer-brain-derived neurotrophic factor (BDNF) (IVOB), and inner-BDNF/outer-VEGF (IBOV) after 4 and 8 weeks of implantation (a), in which the asterisk (*) shows the scaffold fragments and the yellow arrows show vessels (bar: 100 μm); and (b) graph showing the vessel densities in (a) in which the asterisk (*) shows significant differences among the groups at each time point (*p* < 0.05). Adapted from Zhang et al. [274], with permission from American Chemical Society.

It has been suggested that neural defects longer than 5 mm are difficult to recover and regenerate by themselves [266]. With regard to several limitations of autologous and allogenic transplantation of peripheral nerves [267], aligned electrospun nanofibers integrated with growth factors and/or neural stem cells and Schwann cells have been stated to be promising replacements for neurite outgrowth and peripheral nerve regeneration [268,269,270,271,272]. Some studies have focused on neural regeneration triggered by angiogenic electrospun nanofibers [111]. Indeed, the orientation and growth of axons can be guided by aligned nanofibrous matrices in order to allow for the formation of a vascularized network [273]. Zhang et al. investigated the neural regeneration capacity of coaxial electrospun silk fibers loaded with VEGF and brain-derived neurotrophic factor (BDNF) on cavernous nerve regeneration [274]. Two types of core-shell nanofibrous scaffolds were produced and designated as IVOB (inner layer—2.84 × 10^−1^ ng/mg of VEGF/outer layer—5.80 × 10^−1^ ng/mg of BDNF) and IBOV (inner layer—2.84 × 10^−1^ ng/mg of BDNF/outer layer—5.80 × 10^−1^ ng/mg of VEGF). The release profile of BDNF and VEGF from the scaffolds clarified an initial burst at the first 4 days, which was followed by a stable release up to 16 days. To evaluate neovascularization and nerve regeneration capacities, the authors implanted the regenerated silk fibroin (RSF)-neat scaffolds with a length of 9 mm and a width of 2 mm into Sprague–Dawley rats. The histopathological results confirmed more nerve regeneration based on promoted angiogenesis in the inner-BDNF and outer-VEGF scaffolds compared with the inner-EGF and outer-BDNF, owing to faster release of VEGF following 8 weeks post-implantation (Figure 5).

In order to regenerate a vascularized nerve tissue, Xia et al. utilized the dual delivery of NGF and VEGF within the core (via emulsion electrospinning) and sheath (by physical adsorption) of electrospun PLLA nanofibers, in which VEGF had a burst release in the first few days, while NGF could be constantly released for 30 days [275]. In vivo data confirmed the nerve regeneration process followed by neovascularization as modulated by the fibrous scaffolds after 3 months. 

**Table 6:** A summary of experimental studies performed for improving tissue regeneration via applying angiogenic electrospun nanofibrous scaffolds. BDNF, brain-derived neurotrophic factor.

Polymer	Fiber Diameter	Therapeutic Element	Target Tissue	Remarks	Ref.
PLCL	1.16 ± 0.18 μm	Human fibroblast-derived ECM	Skin	Higher proliferation and vascular morphogenesis of HUVECs seeded on the scaffold A promising role on wound healing by increased wound closure rate, mature blood vessel density, and regenerated epidermis and skin appendages after 4 weeks post-implantation	[276]
PLGA		Angiogenin and curcumin	Skin	Maintained bioactivity and sustained release of curcumin and angiogenin in 6 days and 20 days, respectively	[38]
PCL	100 ± 20 nm	Si and Zn ions and CiH	Skin	Releasing Si ions promoted angiogenesis and skin regeneration after 14 days post-implantation Zn ions and ciprofloxacin hydrochloride (CiH) resulted in enhanced hair follicle regeneration and antibacterial activity	[277]
PCL	According to the incubation time with acetone, 2.4 ± 0.7, 1.1 ± 0.3, 0.5 ± 0.1 µm for 10 min, 1 h, and 6 h, respectively	Vasoactive intestinal peptide	Skin	Enhanced cell adhesion and proliferation Promoted wound healing by increased granulation tissue formation and angiogenesis, but not significant re-epithelialization	[278]
Gelatin and PLGA		PEGylated curcumin cobalt nanoparticles	Skin	Enhanced endothelial cell proliferation and VEGF production	[241]
PCL	218.24 ± 35.21 nm	Placental-derived bioactive molecules	Skin	Promoted adhesion, infiltration, and proliferation of fibroblasts and keratinocytes and enhanced vascularization	[163]
Cellulose acetate/gelatin	316 ± 115 nm	Nanohydroxyapa-tite (nHA)	Skin	25 mg nHA loaded in cellulose acetate/gelatin (CA/Gel) showed the highest collagen synthesis, re-epithelialization, neovascularization, and the greatest wound closure value (93.5 ± 1.6%) compared with 12.5 and 50 mg nHA	[279]
PVA and chitosan	716.5 ± 76.1 nm	Desferrioxamine	Diabetic wound	Upregulated expression of HIF-1α, VEGF, and SDF-1α Promoted interaction of fibroblasts and endothelial cells	[18]
Collagen, hyaluronic acid, and gelatin nanoparticles	486 ±151 nm and 534 ±128 nm for HA and COL nanofibers, respectively	VEGF, PDGF, bFGF, and EGF	Diabetic wound	Sustained release of growth factors up to one month owing to encapsulation of the gelatin nanoparticlesThe scaffold possesses similar mechanical properties to native skin	[133]
Heparin mimetic peptide			Diabetic wound	Accelerated wound closure and granulation tissue formation Greater expression of alpha-smooth muscle actin (α-SMA) and VEGF	[259]
Chitosan	50–200 nm		Skin	Greater cell adhesion and cell proliferation, faster regeneration of dermis and epidermis components, and well-vascularization compared with chitosan films and sponges	[254]
Polydioxanone (PDS)	1–17 µm at the concentration of 125 mg/mL PDS	Alginate beads encapsulated with NGF and chondroitinase ABC	Nerve tissue	More cellular infiltration owing to aligned nanofibers using air-gap electrospinning Vascular network formation after 3 weeks post-implantation Regenerating axons following spinal cord injury owing to trophic support and directional guidance of a scaffold	[273]
Poly-L/DL lactic acid (PLA70/30)	657 ± 101 nm for random and 568 ± 81 nm for aligned nanofibers		Damaged brain	Radially aligned nanofibers supported neuronal migration Long-term viability and integration of the newly generated neurons	[111]
Silk fibroin	1.8 ± 0.5 µm	BDNF, VEGF	Nerve tissue	De novo innervation and neovascularization indicated by a positive endothelial marker (von Willebrand factor) and innervation marker (S-100 protein) without inflammation Promoted nerve regeneration and angiogenesis after 8 weeks post-implantation	[272]
PCL/gelatin	400–700 nm	bFGF	Bone	A more controlled release of heparin-mediated bFGF up to 24 h Increased proliferation and migration of hMSCs and tubulogenesis of HUVECs Heparinized nanofibers incorporated with 50 or 100 ng/mL bFGF showed a two- and threefold increase in new bone formation, respectively	[245]
PLA	830.3 ± 211.9 nm and 853.7 ± 238.6 nm for the uncoated and coated fibers, respectively	Surface coating of polydopamine	Bone	Greater ALP activity and osteocalcin of hADSC cultured on the scaffold Up-regulation of ang-1 and vWF proteins	[171]
PCL	580 ± 80 nm	Ceramic nanoparticles including Si^4+^, Ca^2+^, and PO_4_^3-^	Bone	Enhanced bioactivity of PCL nanofibers owing to greater apatite formation Reduced contact angle of PCL-Ca-Si (63° ± 3°) compared with only the PCL scaffold (120° ± 10°)	[48]
PLGA	588.9 ± 110.3 nm	Heparin-mediated immobilization of VEGF Co-culture of HUVECs with human/rat MSCs	Bone	Sustained release of VEGF in conjugation with heparin Enhanced angiogenesis, which was detected by CD31 immunostaining after 3 weeks	[244]
PCL		In situ silica gelation	Bone	Enhanced water wettability and sustained release of silicon ions (28 ppm silicon ions in 14 days) Promoted tubulogenesis of HUVECs Up-regulation of pro-angiogenic markers including CD31, VEGF-A, PDGF-B, and eNOS and osteogenic markers such as COL1 A1, RUNX2, OSTX, and BMP2	[280]
Methylmethacrylate (MMA), hexylmethacrylate (HMA), and (trimethoxysilylprolyl) methacrylate (siMA)	Below 500 nm	Mg implant-coated with electrospun nanofiber containing NO	Bone	Stable and local delivery of NO for targeted tubulogenesis of HUVECs	[281]
PLLA/chitosan	Not mentioned	Icariin, deferoxamine, and polydopamine	Bone	Promoted cell adhesion, proliferation, osteogenic differentiation, and mineralization of MC3T3-E1 through upregulation of Runx-2, ALP, COL 1, and osteocalcin Up-regulation of angiogenic markers of HUVECs including eNOS, HIF-1α, VEGF, and CD31	[43]

## 6. Concluding Remarks and Future Perspectives

The scientific literature shows that some strategies can be potentially used to promote or somehow achieve angiogenesis in tissue substitutes, including different combinations of micro- and nano-biomaterials, stem cells, and bioactive molecules. However, angiogenesis still remains a critical issue in the development of functional engineered tissues and deserves to be investigated more in the future. In this regard, the use of electrospun nanofibrous scaffolds was evaluated as an innovative and highly versatile approach in promoting angiogenesis and subsequent accelerated tissue repair and regeneration. However, conventional electrospun nanofibers have limited potential for cell infiltration and migration, which is considered as a barrier in angiogenesis-promoted wound healing. In order to address this limitation, different strategies have been investigated to increase the pore size of electrospun mats, including melt-electrospinning. In addition, the combination of the electrospinning technique with other methods and technologies (e.g., electrospraying or bioprinting) may be useful to achieve potent angiogenic constructs.

In general, some “geometrical” and physical characteristics of the fibrous mats or scaffolds (e.g., fiber diameter, the wall thickness in the case of hollow fibers, fiber length) can be designed, controlled and finely tailored by acting on the major processing parameters of electrospinning; however, a clear picture is currently lacking on the impact that each of these features may have on angiogenesis. The spatial configuration of fibers in the mats, or in other words, the way in which the fibers are interwoven, can also play a role in many cell activities and responses, like angiogenesis. Furthermore, the composition of basic fibers and the whole construct should be taken into account; although polymers apparently play no significant role in angiogenesis if used alone, polymeric nanofibrous mats can be made pro-angiogenic by embedding bioactive molecules (e.g., growth factors like VEGF) or nano-sized inclusions (e.g., BG nanoparticles releasing metallic cations like Cu^2+^). There is also recent evidence that incorporation of special nanoparticles (e.g., magnetic nanoparticles) in electrospun polymeric nanofibrous constructs can allow one to obtain multifunctional implants with multiple extra-functionalities, including not only angiogenesis, but also the capability of stimulating odontogenesis, thereby disclosing new horizons in the field of periodontal regeneration [282]. The main challenge for the future is perhaps a methodological one, that is, the development of a rational library associating the physico-chemical features of electrospun nanofibrous scaffolds to the following: (i) the processing parameters needed to obtain them (clear and objective reproducibility) and (ii) the biological cues that can be related to their presence/effect, with a special focus on angiogenesis.

## Figures and Tables

**Table 1:** Some of the major angiogenic bioactive molecules that play critical roles in angiogenesis (sorted by time of occurrence) [14]. VEGF, vascular endothelial growth factor.

Bioactive Molecule	Function
VEGF family	Stimulating angiogenesis, permeability, and leukocyte adhesion
Angiopoetin1 (Ang1) and Tie2	Stabilizing vessels and inhibiting permeability
Platelet-derived growth factor-BB (PDGF-BB)	Recruiting of smooth muscle cells (SMCs)
Transforming growth factor-β (TGF-β1)	Stimulating extracellular matrix (ECM) production
Fibroblast growth factor (FGF) and hepatocyte growth factor (HGF)	Stimulating angio/arteriogenesis
Matrix metalloproteinase (MMPs)	Matrix remodeling, release and activation of growth factors
Plasminogen activator inhibitor-1 (PAI-1)	Stabilizing nascent vessels
Nitric oxide synthase (NOS)	Promoting angiogenesis and vasodilation

**Table 2:** Parameters of fibrous scaffolds that may affect angiogenesis.

Parameter	Possible Effect on Angiogenesis	Ref(s)
Porosity	A minimum porosity of 30 to 40 µm is required for metabolite exchange and endothelial cell (EC) entrance	[22,23]
Pore size	Pores greater than 300 µm are required for new blood vessel formation of the constructs	[24,25]
Fiber orientation	Aligned nanofibers promote neovascularization	[26]
Heparin-functionalized nanofibers	Promoting angiogenesis through binding to angiogenic growth factors such as VEGF, HGF, and FGF-2	[27]
Polymer degradation	Slower polymer degradation leads to greater cell mobilization and angiogenesis owing to less acidic environment formation (however, electrospun nanofibrous mats are not mentioned)	[28]
Scaffold stiffness	Greater surface stiffness leads to higher EC spreading and a larger number and greater length of new sprout formation (however, nanofibers are not mentioned)	[29]

**Table 3:** The effects of electrospinning parameters on nanofibers.

Parameters	Effects on Nanofiber Properties	Ref.
*Polymer parameters*		
Polymer concentration	Higher polymer concentration leads to increased fiber diameter and higher pore size and porosity	[21]
Solution viscosity	Increased viscosity results in increased nanofiber uniformity and diameter and reduced beaded morphology	[80]
Molecular weight of polymer	Increased molecular weight leads to higher nanofiber diameter and less bead formation	[81]
Surface tension	Less surface tension leads to proper jet initiation	[82]
Conductivity	Higher solution conductivity leads to decreased fiber diameter	[83]
*Electrospinning parameters*		
Applied voltage	Initially decreases nanofiber diameter and then increases after a point	[84]
Needle tip to collector distance	Too short and too long distances lead to bead formation	[85]
Flow rate	Higher flow rate leads to bead formation, decreased flow rate leads to a decrease in fiber diameter	[86]
Temperature	Higher temperature leads to decreased fiber diameter	[87]
Humidity	Increase in humidity leads to circular pores on the fibers	[88]

**Table 4:** A summary of polymeric nanofibers with the ability to promote angiogenesis. PCL, poly(ε-caprolactone); PLA, poly(lactic acid).

Polymer(s)	Fiber Diameter	Biomedical Application	Remark(s)	Ref.
Expanded 3D PCL nanofiber	Not mentioned	Neovascularization after subcutaneous implantation	Promoted cell infiltration and tissue integration Enhanced regenerative response owing to increased expression of CD68, CCR7, and CD163 markers	[120]
Poly [2-bis-(3 octyloxyphenyl) quinoxaline-5,8-diyl-alt-thiophene-2,5-diyl] (TQ1)	Not mentioned	Angiogenesis	A semiconducting luminescent polymer that could be visualized in situ up to 90 days after subcutaneous implantation using fluorescence imaging Limited inflammation and formation of small capillaries around the fibers	[121]
PLA	657 +/− 101 nm for random and 568+/− 81 nm for aligned nanofibers	Angiogenesis and neurogenesis	Radially aligned nanofibers supported neuronal migration Long-term viability and integration of newly generated neurons	[111]
PHB, PCL, PLA, and PA (polyamide)	Not mentioned	Angiogenesis and cardiac repair	Among the scaffolds used, PHB had the most biocompatibility, biodegradability, angiogenic and potential, as well as expression of pro-inflammatory cytokines including interleukin (IL)-1β, IL-4, IL-6, IL-10, IL-13, and IFN-γ after epicardial implantation	[122]
PCL/collagen/PEO	250 nm +/− 73 nm	Angiogenesis	Lower sprouting vessels in the aligned scaffold, but earlier vascularization in the center of the construct compared with nonwoven scaffolds using microCT scan images	[30]
PDLLA/PCL/gelatin	500–700 nm	Angiogenesis	Anisotropically and heterogeneously aligned scaffold Excellent mechanical properties and bioactivity	[123]
PHB	1603 +/− 73 nm	Angiogenesis and skin reconstruction	Good biocompatibility Advanced properties compared with PCL for skin regeneration Polarization of macrophages to the M2 phenotype	[124]

Abbreviations: PHB: Polyhydroxybutyrate, PEO: polyethylene oxide, PDLLA: Poly(D,L-lactic acid).

**Table 5:** Some examples of experimental studies in which pro-angiogenic factors were integrated into nanofibrous scaffolds. MSC, mesenchymal stem cell; PLGA, poly(lactic-co-glycolic acid); PEG, polyethylene glycol.

Polymer	Pro-Angiogenic Factor	Method of Integration	Remarks	Ref(s)
*Cells*				
PLA/PCL	hUC-MSCs	Seeding cells on the scaffold	PCL significantly increased the angiogenic potential of hUC-MSCs with no additional factors Increased migratory and pro-angiogenic potential	[228]
Polydioxan-one (PDO)	Bone marrow-derived macrophages	Seeding cells on the scaffold	Higher concentrations of polymer led to a larger fiber diameter, pore size, and porosity Increased secretion of pro-angiogenic cytokines including VEGF, bFGF, and TGF-β for the scaffolds with larger pore sizes Pore size of the scaffold is more critical than fiber diameter in macrophage polarization	[21]
PCL/gelatin	Adipose tissue-derived mesenchymal stem cells (ADSCs)	Seeding and coculturing of ADSCs and HUVECs on the scaffolds	Greater sprouting of endothelial cells, formation of a mature blood vessel-like network, and enhanced expression of tight junction proteins	[191]
PCL/gelatin/fi-bronectin	Cardiac progenitor cells (CPC)	Seeding cells on the patches	Electrospun scaffolds maintained durable CPC viability Reduced fibrotic gene expression in rat cardiac fibroblasts Tube formation of HUVECs by media collected from the nanofibrous patches demonstrating the pro-angiogenic potential of the patch	[229]
PLCL/colla-gen nanoyarn fibers	Pig iliac endothelial cells (PIECs) and MC3T3-E1 pre-osteoblastic cells	Seeding cells on nanoyarn scaffold	Formation of complex capillary-like structures after 7 days More cell infiltration into this morphology of electrospun scaffolds compared with conventional nanofibrous electrospun mats	[230]
*Growth factors*				
Gelatin	bFGF	Physical immobilization	Proliferation rate of HUVECs was proportional to the loading concentration of bFGF (0–100 ng/mL) The gradient growth factor distribution effects on vessel direction	[33]
Pullulan/dex-tran nanofibers loaded with fucoidan	VEGF	Physical immobilization	Increased fucoidan content led to increased retention of VEGF bioactivity and angiogenic response up to 14 days Promoted cellular infiltration and complete biodegradation of the construct up to 7 days after subcutaneous implantation in mice	[133]
Poly(estera-mide) PEA	FGF2, FGF9	Emulsion electrospinning	Sustained release and preserved bioactivity of FGF2 and FGF9 over 28 days Enhanced tubular formation	[35,231,232]
PLGA	Collagen containing VEGF	Surface coating	Improved pre-vascularization of the construct after seeding HUVECs Anastomose formation between the implanted construct and mice vasculature confirmed by immunostaining of CD31 and von Willebrand factor	[233]
*Others*				
PEG	bFGF and VEGF-encoding plasmids	Coaxial electrospinning	Improved cell viability and attachment and extracellular secretion of collagen Ⅳ and laminin Alleviated inflammation reaction and enhanced microvessel generation	[234]
Nylon	Insulin		Outer porous layer supports cellular infiltration and vascularization Inner low porosity layer supports cellular isolation Localized cell transplantation	[235]
PCL	Heparin and VEGF	Immersing in heparin and VEGF solution, respectively	Stimulated neovascularization with minimum immunological rejection	[236]
PLCL	Substance P		Sustained release of substance P up to 30 days Improved host cell infiltration, blood vessel formation, and MSC recruitment in vivo Existence of laminin-positive blood vessels and von Willebrand factor cells	[237]
PCL	Vitamin D3	Blending	Promoted cellular infiltration and neovascularization Reduced inflammation and infection	[238]
Silk fibroin/gelatin	Astragaloside IV		Accelerated wound healing and prevented scar formation by stimulating wound closure and increasing angiogenesis in partial-thickness burn wounds	[239]
PCL	Collagenase	Surface immobilization	Collagenase (0.01 mg/mL) promoted smooth muscle cell (SMC) migration Enhanced capillary formation after subcutaneous implantation	[240]
Gelatin/PLGA	Cobalt and PEGylated curcumin	Core-shell electrospinning	Promoted EC patterning and enhanced VEGF production	[241]
PLLA/chitosan nanofibers coated with polydopa-mine	Icariin and deferoxamine	Surface modification	Synergistic effects on osteogenesis and angiogenesis	[43]
